# Smart Manipulation of Complex Optical Elements via Contact‐adaptive Dry Adhesives

**DOI:** 10.1002/advs.202303874

**Published:** 2023-09-08

**Authors:** Shuai Li, Hongmiao Tian, Chunhui Wang, Xiangming Li, Xiaoliang Chen, Xiaoming Chen, Jinyou Shao

**Affiliations:** ^1^ Micro‐ and Nano‐technology Research Center State Key Laboratory for Manufacturing Systems Engineering Xi'an Jiaotong University Xi'an Shaanxi 710049 China; ^2^ Frontier Institute of Science and Technology (FIST) Xi'an Jiaotong University Xi'an Shaanxi 710049 China

**Keywords:** biomimetic dry adhesives, complex optical elements, contamination‐free manipulation, smart manipulation, stiffness modulation

## Abstract

The implementation of complex, high‐precision optical devices or systems, which have vital applications in the aerospace, medical, and military fields, requires the ability to reliably manipulate and assemble optical elements. However, this is a challenging task as these optical elements require contamination‐free and damage‐free manipulation and come in a variety of sizes and shapes. Here, a smart, contact‐adaptive adhesive based on magnetic actuation is developed to address this challenge. Specifically, the surface bio‐inspired adhesives made of fluororubber facilitate contamination‐free and damage‐free adhesion. The stiffness modulation of packaged magnetorheological grease based on the magnetorheological effect endows the smart adhesive with a high conformability to the optical elements in the soft state, a high grip force in the stiff state, and the ability to quickly release the optical elements in the recovered soft state. The smart adhesive provides a versatile solution for reliably and quickly manipulating and assembling multiscale optical elements with planar or complex 3D shapes without causing surface contamination or damage. These extraordinary capabilities are demonstrated by the manipulation and assembly of various optical elements, such as convex/concave/ball lenses and extremely complex‐shaped light guide plates. The proposed smart adhesive is a promising candidate for conventional optical element manipulation technologies.

## Graphical abstract

The proposed smart bio‐inspired contact‐adaptive dry adhesive, with switchable and contamination‐free adhesion, enables reliable and fast manipulation and assembly of planar or complex‐shaped optical elements with sizes ranging from a few millimeters to tens of centimeters via magnetic actuation, opening new application avenues for manipulation and assembly of optical elements and even space telescopes in space.

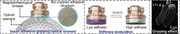

## 1 Introduction

The reliable manipulation and assembly of optical elements are essential tasks for obtaining complex and high‐precision optical devices or systems, which have important applications in many fields, including the aerospace, medicine, and military fields. For example, the precise installation of Webb's primary mirror segments onto the telescope structure has been the predominant factor contributing to the successful operation of NASA's Webb Telescope.^[^ ^1^ ^]^ Furthermore, the effective operation of the FAST (Five‐hundred‐meter Aperture Spherical radio Telescope), the world's largest filled‐aperture radio telescope, is largely due to the precise assembly of its active primary surface, which has a diameter of 300 m and is made up of 4500 panels.^[^ ^2^ ^]^ However, certain requirements, such as the ability to manipulate optical elements without causing any contamination or damage to their surface as well as the capacity to handle optical elements of various sizes and shapes, including spherical, concave, convex, planar, and specially shaped surfaces, make the manipulation and assembly tasks extremely challenging.

For the manipulation and assembly, one of the most common procedures is to use a multifinger design mimicking the gripping/releasing of the human hand.^[^ ^3^ ^]^ A large number of optimization schemes for such multifinger designs have been proposed, which offer considerable potential for the manipulation and assembly of objects of diverse shapes.^[^ ^3^, ^4^ ^]^ However, this active approach usually entails a visual/force feedback as well as complex auxiliary devices and algorithms to determine the optimal manipulation schemes, which makes the whole system cumbersome.^[^ ^4^, ^6^ ^]^ Additionally, multifinger designs that rely on mechanical clamps are incapable of dealing with thin and fragile objects, such as optical elements, resulting in possible damage to their surface.^[^ ^3^, ^7^ ^]^ On the other hand, passive suction‐based gripper designs, which are used in large‐scale industry production, reduce the system complexity and may thus provide a promising solution.^[^ ^8^ ^]^ However, while being suitable for planar and thick objects, such suction‐based grippers become problematic when maneuvering objects with complex geometries or fragile features, such as optical elements, because of the possible air leakage at their projecting and concave corners or the possibility to cause surface damage due to stress concentration at the contact interface. Thus, due to the abovementioned difficulties, the manipulation and assembly of optical elements have always been challenging. Therefore, the development of a universal manipulation method that can reliably handle optical elements of various shapes and sizes without causing any damage to their surface is essential.

To this end, biomimetic dry adhesion, which is based on the van der Waals interactions^[^ ^9^, ^10^ ^]^ rather than clamping (friction) or suction, represents a good strategy.^[^ ^11^, ^12^, ^13^, ^14^, ^15^ ^]^ Owing to the fact that the interface stress is averaged, the utilization of dry adhesion prevents unwanted stress concentration at the contact areas,^[^ ^15^, ^16^, ^17^ ^]^ which drastically reduces the possibility to damage or even fracture thin or fragile objects compared with suction‐based manipulation approaches. Furthermore, the dry adhesion induced by van der Waals interactions, which is still valid in a vacuum, is also suitable for the manipulation of objects in space, such as the optical lenses of space telescopes.^[^ ^18^, ^19^ ^]^ The majority of existing research works on dry adhesion have focused on achieving a high adhesion of dry‐adhesion structures on target objects;^[^ ^13^, ^20^, ^21^, ^22^, ^23^, ^24^, ^25^, ^26^, ^27^, ^28^, ^29^, ^30^, ^31^ ^]^ adhesion strengths exceeding 150 kPa on smooth surfaces have been reported, surpassing the performance of the gecko's foot adhesion on such surfaces.^[^ ^9^ ^]^ However, these dry‐adhesion structures commonly disregard the bad effect on the target surfaces, i.e., they would most likely cause the contamination of materials to the target surfaces during the repeated gripping/releasing operations, as confirmed by our experiments in the present work. Hence, current dry‐adhesion structures are not capable of handling optical elements that require to remain contamination‐free, and this results in a sharp decrease in the imaging precision of optical devices or systems after assembly.

In addition, previous studies on dry adhesion have mainly addressed the adhesion requirements for a successful manipulation of planar or low‐curvature objects, i.e., strong gripper/device adhesion for picking and weak gripper/device adhesion for releasing, through external stimuli, such as heating,^[^ ^32^, ^33^ ^]^ light irradiation,^[^ ^27^, ^34^ ^]^ voltage application,^[^ ^35^, ^36^ ^]^ electrostatic interaction,^[^ ^37^ ^]^ magnetic field application,^[^ ^38^, ^39^ ^]^ or preloading.^[^ ^40^, ^41^ ^]^ Such dry adhesion, however, is poorly tailorable for optical elements with complex 3D surfaces. Although some adhesion designs exploiting either the volume change of the cavity based on the elastic energy storage,^[^ ^42^, ^43^ ^]^ or the interfacial equal load sharing,^[^ ^18^, ^23^ ^]^ or the modulus modulation of shape‐memory polymers (SMPs)^[^ ^28^, ^44^ ^]^ or PDMS‐based adhesives^[^ ^45^, ^46^ ^]^ are capable of dealing with the gripping/releasing of objects of diverse shapes and sizes, numerous limitations still remain, such as the requirements for complex auxiliary devices, and tight sealings and the poor manipulation capabilities for planar surfaces as well as the slow response times and potential material contamination of the target surfaces during the repeated gripping/releasing operations. Thus, the main challenge for optical elements with both planar and complex 3D surfaces lies in the ability to achieve manipulation (gripping and releasing) and assembly quickly and simply without causing any surface contamination or damage.

Motivated by the bio‐inspired adhesives and smart magnetorheological materials, this work aims to provide a universal, smart, contact‐adaptive adhesive that can quickly manipulate and assemble planar or complex‐shaped optical elements of sizes ranging from a few millimeters to tens of centimeters without causing any surface contamination or damage. The proposed smart adhesive combines a bio‐inspired adhesive structure with a packaged smart magnetorheological grease. The bio‐inspired adhesive structure is capable of attaining contamination‐free and damage‐free adhesion. The stiffness modulation of the smart magnetorheological grease by a magnetic field can give the smart adhesive high conformability to arbitrarily shaped optical elements in the soft state and high interfacial fracture resistance capable of sustaining a high grip force in the stiff state. Additionally, as the stiffness modulation of the magnetorheological grease is triggered via the application of a magnetic field, the response time of the smart adhesive is on the scale of seconds (≈3 s), which is considerably shorter than that of thermally responsive structures, such as SMP grippers. Consequently, the proposed smart adhesive can reliably and rapidly handle optical elements with diverse shapes and sizes without causing any surface contamination or damage by only switching on a magnetic field. These findings open new application avenues for the manipulation and assembly of optical elements, and even space telescopes in space.

## 2 Results and Discussion

### 2.1 Design and Operation Principles of the Proposed Smart Adhesive

**Figure 1:**
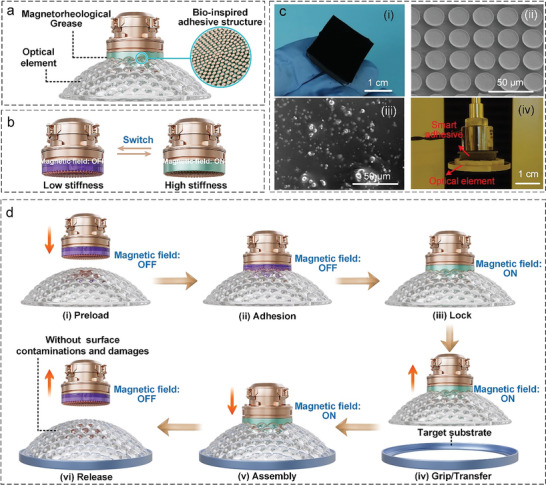
Design and operation principles of the proposed smart adhesive. a) Schematic of the smart adhesive (consisting of the bio‐inspired adhesive structure on the surface and the packaged smart magnetorheological grease inside the sealing cavity) gripping an optical component with a complex 3D surface. b) Schematic of the stiffness modulation of the smart magnetorheological grease through the application of a magnetic field. The light purple color represents low stiffness, while the light green color represents high stiffness. c) (i) Photograph of the smart adhesive, (ii) SEM image of the bio‐inspired adhesive structure made of FKM, (iii) SEM image of the embedded magnetorheological grease made of PDMS base and carbonyl iron powder with a particle size of 3.6 µm, and (iv) demonstration of the ability of the smart adhesive to reliably grip an optical element with a complex 3D surface under application of a magnetic field. d) Schematic of the manipulation and assembly process of an optical element using the smart adhesive. (i) and (ii) The initially soft smart adhesive approaches the optical element and comes into contact with it. (iii)–(v) Then, the smart adhesive becomes stiff under application of a magnetic field to ensure the reliable locking, gripping, transferring, and assembling of the optical element. (vi) Finally, upon removal of the magnetic field, the smart adhesive recovers its initial soft state, which allows the release of the optical element due to interfacial crack progression caused by its own weight or other auxiliary devices.

The proposed smart adhesive is shown in **Figure** **1**a. Specifically, the bio‐inspired adhesive structure can achieve contamination‐free and damage‐free adhesion by using fluororubber (FKM) instead of the commonly used multicomponent crosslinked polymers (a detailed explanation will be provided in next section). The stiffness of the smart magnetorheological grease can be modulated by a magnetic field based on the magnetorheological effect (as depicted in Figure 1b), endowing the smart adhesive with a high conformability to arbitrarily shaped optical elements in the soft state and a high interfacial fracture resistance, which can sustain a high grip force, in the stiff state. The smart adhesive was fabricated using an embedding method (see Section S1 and Figure S1, Supporting Information), which ensures the sufficient sealing of the design and prevents the leaking of the magnetorheological grease during the manipulation process. Figure 1c(i) shows a photograph of the smart adhesive (size: 20 × 20 × 4.2 mm^3^), Figure 1c(ii) shows the bio‐inspired adhesive structure on the surface, and Figure 1c(iii) shows the magnetorheological grease inside the sealing cavity. The smart magnetorheological grease (see Section S2 and Figure S2, Supporting Information), which plays a crucial role in the stiffness modulation, is made of a matrix material (PDMS(Polydimethylsiloxan) base) and suspended particles (carbonyl iron powder with a particle size of 3.6 µm), as shown in Figure 1c(iii), where the shiny white dots are the incorporated carbonyl iron particles. Exploiting the stiffness modulation of the magnetorheological grease (the stiffness increases after contact between the smart adhesive and an irregular object), the smart adhesive is capable of reliably holding optical elements with a complex 3D surface, as shown in Figure 1c(iv).

Figure 1d depicts the whole manipulation and assembly process of an optical element with a 3D surface. Initially, the soft smart adhesive approaches the optical element until it comes into contact with it and conforms to it under a preload force (Figure 1d(i),(ii)). Then, the smart adhesive is stiffened through the application of a magnetic field to lock the optical element. The grip force is large due to the high interfacial tear‐resistant strength originating from the high energy storage and the interface van der Waals forces; thus, the optical element can be easily gripped, transferred, and assembled (Figure 1d(iii)–(v)). After the removal of the magnetic field, the smart adhesive recovers its original soft state, which enables the release of the optical element via interfacial crack progression caused by its own weight or other auxiliary devices (Figure 1d(vi)).

### 2.2 Preparation of the Bio‐Inspired Adhesives and Verification of their Non‐Contamination Properties

**Figure 2:**
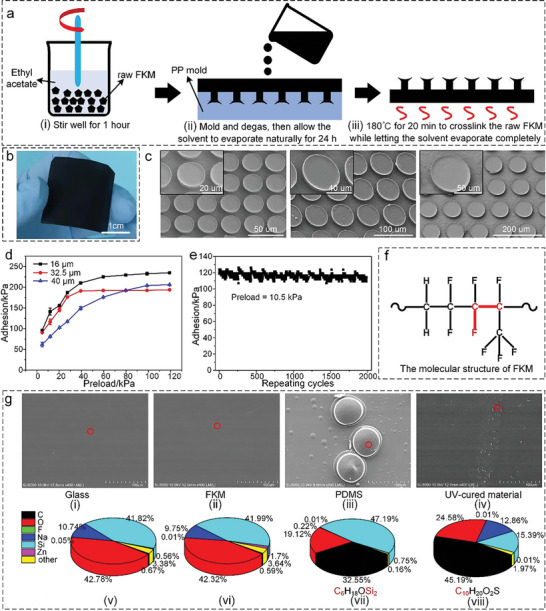
Preparation and characterization of the bio‐inspired adhesives and verification of their non‐contamination properties. a) Preparation process of the bio‐inspired adhesives with FKM. (i) Stir thoroughly for 1 h. (ii) Mold and degas, then allow the solvent to evaporate naturally for 24 h. (iii) Place the film in an oven at 180 °C for 20 min to crosslink the raw FKM while letting the solvent evaporate completely. b) Photograph of a bio‐inspired adhesive film with an area of 3 × 3 cm^2^. c) SEM images of the bio‐inspired adhesive microstructures with three different radii (16, 32.5, and 40 µm). d) Comparison of the adhesion of the three bio‐inspired adhesives on flat glass as a function of the preload. e) Repeatability tests of the bio‐inspired adhesives (microstructure radius of 16 µm). f) Molecular structure of FKM. g) Comparison of the contamination left on glass surfaces by bio‐inspired adhesives made of three different materials (FKM, PDMS, and a UV‐cured material) using (i)–(iv) SEM and (v)–(viii) EDS. Error bars denote ±SD.

**Figure** **2a** shows the preparation process of the bio‐inspired adhesives, in which FKM rather than the conventional multicomponent crosslinked polymers is used as the raw material to prepare the bio‐inspired adhesives due to its superior non‐contamination properties and fatigue durability (the detailed explanation will be provided below).^[^ ^47^, ^48^ ^]^ The original form of FKM is solid, so it needs to be dissolved by ethyl acetate before it can be used for casting and molding. After thorough stirring for 1 h (Figure 2a(i)), the fully dissolved raw FKM was poured into a polypropylene (PP) mold (see Section S3 and Figure S3, Supporting Information) with inverted bio‐inspired adhesive structures. It should be mentioned that the reason for selecting the PP material for the mold is that this crystalline plastic is hard to dissolve in ethyl acetate. Subsequently, the PP mold covered by the dissolved FKM was degassed in a vacuum chamber and left standing for 24 h to evaporate the solvent (Figure 2a(ii)). Finally, the demolded bio‐inspired adhesive film was placed in an oven at 180 °C for 20 min to induce the crosslinking reaction of the raw FKM while letting the solvent evaporate completely (Figure 2a(iii)). The obtained large‐area, bio‐inspired adhesive film (with an area of 3 × 3 cm^2^) is shown in Figure 2b; it comprises dense microstructures on the surface.

Figure 2c; Figure S4 (Supporting Information) show the SEM (Scanning Electron Microscope) images of the bio‐inspired adhesive microstructures with three radii (16, 32.5, and 40 µm); it can be seen that they exhibit good uniformity and consistency. To quantify the adhesion force of the bio‐inspired adhesives, preloading and pulling‐up tests were carried out at a speed of 1 mm/min (see Section S4 and Figure S5a, Supporting Information). As shown in Figure 2d, with the increase in the preload, the adhesion of all three adhesives (with microstructure radii of 16, 32.5, and 40 µm) on flat glass increases until it reaches saturation values at a preload of 99 kPa (233, 193, and 204 kPa, respectively). The adhesion mechanism of the biomimetic adhesives was discussed in our previously published research,^[^ ^49^ ^]^ which is the combination of van der Waals force and suction stress in an air environment, and the relationship between interfacial adhesion strength and biomimetic adhesives with different radii was also discussed from the perspective of numerical analysis. The reusability of the bio‐inspired adhesives with a microstructure radius of 16 µm was also investigated, as shown in Figure 2e; Figure S6 (Supporting Information). It was found that after 2000 attachment and detachment cycles at a preload of 10.5 kPa, the bio‐inspired adhesives could still maintain a high adhesion strength within the interval 107.14‐126.19 kPa (no decrease was observed, and the reason for the undulated or periodic repeating adhesion data is that the sampling frequency (50 Hz) of the force test equipment we used is not high enough. It is not guaranteed that the maximum adhesion point will be captured each time, but rather in the periodic interval around it), which is opposite to the case of bio‐inspired adhesives made of PDMS that we studied in a previous work (we observed a decrease of 10.6% after 120 attachment and detachment cycles at a preload of 15 kPa).^[^ ^49^ ^]^ Figure S7 (Supporting Information) presents the comparative SEM images before and after the tests, no damage, scratch or fracture on the surface of the structure is observed after 2000 repeated grip and release tests, demonstrating the excellent scratch resistance of the prepared bio‐inspired adhesives. This extraordinary fatigue durability and scratch resistance of the prepared bio‐inspired adhesives are mainly due to the special molecular structure of FKM (Figure 2f). The energy of the C–F bond in FKM is higher (485 KJ·mol^−1^) than that of other carbon bonds because the carbon atoms on the main/side chain are connected with highly electronegative fluorine atoms (see Section S5 and Table S1, Supporting Information). Furthermore, the covalent radius of the fluorine atoms is 0.64 Å, which is equivalent to the half length of the C–C bond (1.34 Å), which implies that the fluorine atoms may effectively shield the C–C main chain and maintain its stability. The combined effects of the high C–F bond energy and the short covalent radius of the fluorine atoms in FKM result in the observed excellent fatigue durability and robustness of the bio‐inspired adhesives, which also do not exhibit any trace of contamination.

Figure 2g shows representative results of the contamination tests performed on the bio‐inspired adhesives made of FKM and other two types of frequently‐used materials for dry adhesion (PDMS and an ultraviolet (UV)‐cured material). Clearly, the bio‐inspired adhesives made of FKM leave no contamination on the tested glass surface (compared with the factory glass surface) after 2000 attachment and detachment cycles; this is in stark contrast to the glass surface after coming into contact with the bio‐inspired adhesives made of PDMS and the UV‐cured material, which exhibits noticeable contamination (fractured microstructures or residual material), as can be seen from the SEM images (Figure 2g(i)–(iv)). These results are confirmed by the energy‐dispersive spectroscopy (EDS) surface analysis conducted on areas of possible contamination on the tested glass surfaces (the red circles in the SEM images)), which provides additional evidence of the differences between the bio‐inspired adhesives made of FKM, PDMS, and UV‐cured materials regarding contamination (Figure 2g(v)–(viii)). Excluding the possible elements introduced during the manufacturing process of the different glass pieces, the element content of the glass surface after contact with the bio‐inspired adhesives made of FKM is basically unchanged with respect to that of the factory glass surface. However, after contact with the bio‐inspired adhesives made of PDMS and the UV‐cured material, the element content of the glass surface changes noticeably. For example, the content of the C and Si elements of the glass surface after contact with the PDMS bio‐inspired adhesive is 32.55% and 47.19%, respectively, which is significantly higher than that measured for the factory glass surface (0.67% and 41.82%, respectively). Similarly, after contact with the bio‐inspired adhesive made of the UV‐cured material, the C element content of the glass surface is 45.19%, which is considerably higher than that of the factory glass surface. In addition, the increased element content on the contaminated areas of the tested glass surfaces is the same as that of the most abundant elements of the materials used, such as the C and Si elements of PDMS (main component: C_6_H_18_OSi_2_) and the C element of the UV‐cured material (main component: C_10_H_20_O_2_S). Hence, it can be concluded that bio‐inspired adhesives based on PDMS or UV‐cured materials contaminate the object they come into contact with during the repeated gripping/releasing operations, while this is not the case for the proposed FKM‐based bio‐inspired adhesive.

### 2.3 Mechanism of the Magnetically actuated Stiffness Modulation of the Smart Adhesive

**Figure 3:**
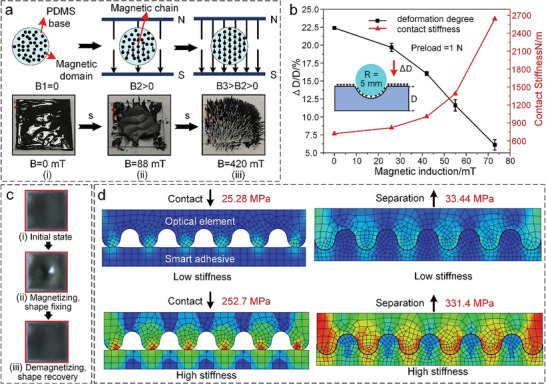
Mechanism of the stiffness modulation of the smart adhesive. a) Illustration of the stiffness modulation of the magnetorheological grease based on the magnetorheological effect. b) Variation in the deformation degree and contact stiffness of the smart adhesive under the action of a magnetic field. c) Actual stiffness modulation effect of the smart adhesive. (i) Initial soft and flat state of the smart adhesive. (ii) Fixed shape of the smart adhesive after embedding the spherical probe, applying the magnetic field, and then removing the spherical probe. (iii) Shape recovery of the smart adhesive after demagnetization. d) Illustration of the stiffness modulation effect of the smart adhesive on its conformability and adhesive force for optical elements with a complex 3D surface using the FEA. Error bars denote ±SD.

The stiffness modulation of the smart adhesive is achieved through the dispersion and clustering of magnetic domains (the incorporated carbonyl iron powder particles) in the magnetorheological grease based on the magnetorheological effect,^[^ ^50^ ^]^ as shown in **Figure** **3**a. In the absence of an external magnetic field, the magnetic domains are arranged randomly in the matrix (Figure 3a(i)), which leads to a low apparent viscosity and a low shearing stiffness (namely, the low‐stiffness state of the smart adhesive). Once the magnetic field is activated, the magnetic domains are reoriented and form magnetic chains along the direction of the magnetic field within seconds (see Figure 3a(ii): A small number of magnetic chains are formed when the magnetic field is 88 mT). As the magnetic field continues to increase, an increasing number of magnetic chains are generated and become stiffer (see Figure 3a(iii): Almost all magnetic chains are activated when applying a magnetic field of 420 mT), resulting in the high apparent viscosity and high shearing stiffness of the magnetorheological grease (that is, the high‐stiffness state of the smart adhesive). Based on the Bingham fluid model, the relationship between the shearing stiffness of the magnetorheological grease and the applied magnetic field can be expressed as *τ_y_* = *αH* ^2^ at a low magnetic field,^[^ ^51^, ^52^, ^53^ ^]^ where *τ_y_* represents the shear yield stress, which is positively correlated with the shearing stiffness, *α* is linked to the magnetic susceptibility of the magnetorheological grease and its volume fraction, and *H* represents the magnetic field (see Section S6, Supporting Information). This equation reveals that the shear yield stress (shearing stiffness) of the magnetorheological grease is proportional to the magnetic field squared.

The hysteresis loop of the magnetorheological grease is shown in Figure S8 (Supporting Information). The magnetorheological grease can exhibit excellent magnetization properties with 123.46 A·m^2^·kg^−1^ magnetization at 800.83 kA·m^−1^ magnetic field strength, which is capable of inducing high shear yield stress. The coercive force and remanence of the magnetorheological grease are 0.8528 kA·m^−1^ and −0.66269 A·m^2^·kg^−1^, respectively, demonstrating that the hysteresis of the magnetorheological grease is very weak. Figure S9 (Supporting Information) shows the viscoelastic response of the magnetorheological grease. It can be seen that the viscosity of the magnetorheological grease exhibits an obvious shear thinning from 14 200 Pa·s at 0.1 s^−1^ shear rate to 63.2 Pa·s at 104 s^−1^ shear rate. Besides, the magnetorheological grease behaves similarly to a viscous liquid because its loss modulus is much higher than the storage modulus at various strains and constant 5 Hz frequency. In addition, the effect of the dispersed state of the magnetic particles in the magnetorheological grease on its response frequency and cycle stability is also discussed, as shown in Figure S10 and S11 (Supporting Information). After applying a magnetic field of 200 mT, it is obvious that the magnetorheological effect of the magnetorheological grease with stirring for 1 h is slightly better than that with stirring for 1 min at the same response time from Figure S10 (Supporting Information) due to the better dispersed state of the magnetorheological grease with stirring for 1 h (Movie S1–S2, Supporting Information). In the 10 cycling tests (Figure S11, Supporting Information), the magnetorheological effect of the magnetorheological grease with stirring for 1 h is always stable (Movie S3, Supporting Information). However, the magnetorheological effect of the magnetorheological grease with stirring for 1 min performs poorly in the first few cycles and better and better in the last few cycles due to the more uniform dispersed state induced by the magnetorheological effect (Movie S4, Supporting Information).

Using a spherical probe with a 10‐mm diameter pressed against the magnetically actuated smart adhesive (inset), Figure 3b demonstrates the effect of the magnetic field on the deformation degree and contact stiffness of the smart adhesive. Under a preload force of 1 N, the deformation degree of the smart adhesive *ΔD*/*D* (where *ΔD* is the pressing depth of the spherical probe, and *D* is the thickness of the smart adhesive) decreases with increasing magnetic induction. When no external magnetic field is applied, the maximum *ΔD*/*D* (≈22.5%) is obtained, indicating that the smart adhesive is soft. When a magnetic field of 73 mT is applied, the smart adhesive has a minimum *ΔD*/*D* of 6.15%, showing that it is stiff (hard to be embedded). The corresponding contact stiffness, *K* = *F*/*ΔD* (where *F* is the preload force), which is negatively correlated with the deformation degree *ΔD*/*D*, increases with increasing magnetic induction (see Section S4 and Figure S5b, Supporting Information). The contact stiffness of the smart adhesive reaches 2650 N·m^−1^ for a magnetic field of 73 mT, which is a factor of 5.9 greater than that of the adhesive in the absence of magnetic stimulation. This marked increase in contact stiffness demonstrates the modulation effect of the magnetic field on the stiffness of the smart adhesive and, by extension, on the adhesive force for handling optical elements. The actual stiffness modulation of the smart adhesive is shown in Figure 3c. It is clear that, in the absence of the magnetic field, the smart adhesive is in a soft and flat state (Figure 3c(i)). However, after using a spherical probe to press into the surface of the smart adhesive, applying a magnetic field, and subsequently removing the spherical probe, the smart adhesive maintains a conformal shape because it is now in the stiff state (Figure 3c(ii)). After switching off the magnetic field, the smart adhesive recovers its initial soft and flat state (Figure 3c(iii)).

Numerical simulations based on the finite element analysis (FEA) were implemented to further demonstrate the stiffness modulation effect of the smart adhesive on its conformability and adhesive force for optical elements with a complex 3D surface, as sketched in Figure 3d. During the contact and separation processes, the optical element was assumed to be pushed and pulled by a preset 0.01‐mm displacement, and the elastic modulus of the high‐stiffness smart adhesive was set to be 10 times greater than that of the low‐stiffness smart adhesive. Specifically, the highest interfacial von Mises stress of the low‐stiffness smart adhesive in contact with an optical element with a 3D surface was 25.28 MPa, whereas it was 252.7 MPa for the high‐stiffness smart adhesive. This indicates that the backing layer of the low‐stiffness smart adhesive can be easily embedded using the same pressing force as that of the high‐stiffness smart adhesive, implying that its conformability to an optical element with a 3D surface is superior. In addition, after the low‐ and high‐stiffness smart adhesives conformed to the optical element, the highest interfacial Mises stresses were 33.44 and 331.4 MPa during the separation process, respectively. This means that the interfacial tear‐resistant strength of the high‐stiffness smart adhesive is higher, implying that a greater pull‐up force is required to separate the optical element and the high‐stiffness smart adhesive, which leads to a higher pulling‐off force and vice versa. According to previous works,^[^ ^54^, ^55^ ^]^ the adhesive force *F_c_* between a polymer adhesive and a rigid surface is related to the compliance of the adhesive *C* and the true interfacial contact area *A* as *F* *_c_* ~(*A*/*C*)^1^. For the contact process, the smart adhesive is soft (low‐stiffness state) and can conform better to the 3D surface of the optical element, which increases *A*. For the gripping process, the smart adhesive becomes stiff (high‐stiffness state) and exhibits an enhanced interfacial tear‐resistant strength, which reduces *C*. The combined effects of the increased *A* and the decreased *C* result in a greater adhesive force *F_c_*. For the release process, the smart adhesive recovers its soft state, and the interfacial tear‐resistant strength is sharply reduced, which increases *C*. The increase in *C* outweighs the increase in *A*, resulting in a lower *F_c_*; hence, the optical element can be easily released due to its own weight or with the aid of auxiliary devices.

### 2.4 Adhesion Characterization of the Smart Adhesive

**Figure 4:**
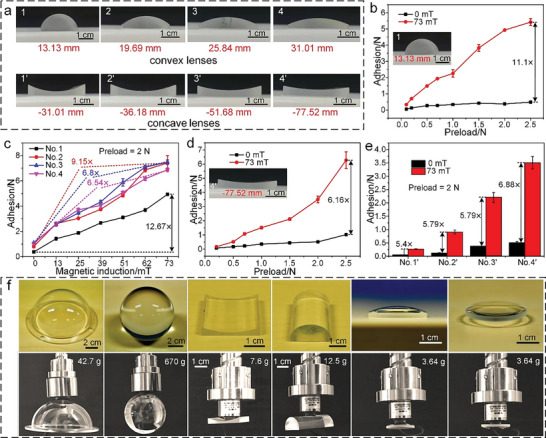
Adhesion characterization of the smart adhesive on optical elements with various shapes based on stiffness modulation. a) Some typical optical elements chosen for the adhesion tests, namely convex lenses (Nos. 1–4, with curvature radius of 13.13, 19.69, 25.84, and 31.01 mm, respectively) and concave lenses (Nos. 1′–4′, with curvature radius of −31.01, −36.18, −51.68, and −77.52 mm). b) Comparison of the adhesive force of convex lens No. 1 in the absence of the magnetic field and under a magnetic field of 73 mT (as a function of the preload). c) Influence of the magnetic field on the adhesive force for convex lenses Nos. 1–4 at a 2‐N preload. d) Comparison of the adhesive force of concave lens No. 4′ in the absence of the magnetic field and under a magnetic field of 73 mT (as a function of the preload). e) Influence of the magnetic field (0 and 73 mT) on the adhesive force of concave lenses No. 1′–4′ at a 2‐N preload. f) Illustration of the proposed smart adhesive reliably holding optical elements with various shapes, such as an optical dome, a ball lens, a concave lens, a convex lens, and a meniscus lens. Error bars denote ±SD.

To quantify the adhesive force of the smart adhesive for optical elements with various shapes, some typical optical elements, such as convex and concave lenses with varying curvatures (**Figure** **4**a), were selected for testing utilizing a conventional preload–pause–pull‐up method at a speed of 1 mm·min^−1^ (see Section S4 and Figure S5c, Supporting Information). Figure S12a (Supporting Information) shows the force‐time curves obtained for the smart adhesive (size: 20 × 20 × 4.2 mm^3^) on convex lens No. 1 (inset, curvature radius of 13.13 mm) under increasing magnetic field and a 2‐N preload force; it should be noted that the magnetic field was only applied during the pause and pull‐up stages because the preload stage requires a high conformability of the smart adhesive to the convex lens. Clearly, as the magnetic field increases, the adhesive force also increases. Figure 4b illustrates the adhesive force of the smart adhesive on convex lens No. 1 as a function of the preload both in the absence of the magnetic field and under a magnetic field of 73 mT. At a given magnetic field, the adhesive force increases with increasing preload, and at a 2.5‐N preload, the adhesive force is 5.422 N under a 73‐mT magnetic field, which is a factor of 10.1 higher than that obtained (0.49 N) in the absence of the magnetic field. This demonstrates the contribution of the magnetorheological grease stiffness modulation to the extraordinary adhesion capability of the smart adhesive. Figure 4c shows how an increase in the magnetic field affects the adhesive force for convex lenses Nos. 1–4 (with curvature radii of 13.13, 19.69, 25.84, and 31.01 mm, respectively) at a 2‐N preload. The adhesive force increases with increasing magnetic field, and as the curvature of convex lens No. 1 is greater than that of the other three lenses, its contact area with the smart adhesive in the test is smaller for a given preload, resulting in a slightly lower adhesion force. Furthermore, the curvature radii of convex lenses Nos. 2–4 are similar to or greater than the side length of the smart adhesive (20 mm); thus, the contact areas of the three lenses with the smart adhesive in the test are similar, resulting in a similar adhesive force. Under a magnetic field of 73 mT, the adhesive force on convex lenses Nos. 1–4 can reach 4.94, 7.41, 7.48, and 6.87 N, respectively, which is around 12.67, 9.15, 6.8, and 6.54 times the values of 0.39, 0.81, 1.1, and 1.05 N measured in the absence of the magnetic field, respectively. It can thus be inferred that the greater the curvature of the convex lens, the more noticeable the enhancement in the adhesive force, and this enhancement will continue to increase upon applying a higher magnetic field.

The results of multiple adhesion tests for the smart adhesive on concave lens No. 4′ (inset, curvature radius of −77.52 mm) under the action of the magnetic field are shown in Figure 4d; Figure S12b (Supporting Information). It is clear that, for a given magnetic field (0 or 73 mT), the adhesive force increases as the preload increases. At a 2.5‐N preload, the adhesive force on concave lens No. 4′ is 6.28 N (under a magnetic field of 73 mT), which is more than 5.16 times the value of 1.02 N measured in the absence of the magnetic field. Regarding concave lenses Nos. 1′–4′ (with curvature radii of −31.01, −36.18, −51.68, and −77.52 mm), the adhesive force for a 2‐N preload both in the absence of the magnetic field and under a magnetic field of 73 mT is illustrated in Figure 4e. Regardless of whether the magnetic field is applied or not, the adhesive force decreases from concave lens No. 4′ to concave lens No. 1′. This is primarily due to the increasing curvature from concave lens No. 4′ to concave lens No. 1′, which results in a smaller contact area with the smart adhesive during the test. The adhesive force on concave lenses Nos. 1′–4′ is 0.27, 0.91, 2.2, and 3.51 N, respectively, under a magnetic field of 73 mT, which is approximately 5.4, 7.58, 5.79, and 6.88 times the values of 0.05, 0.12, 0.38, and 0.51 N measured in the absence of the magnetic field, respectively. Figure S12c,d (Supporting Information) illustrate the effect of the magnetic field on the adhesive force for a spherical lens with a diameter of 8 cm. The adhesive force under a magnetic field of 73 mT is as high as 7.25 N at a 2.5‐N preload, which is more than 6.55 times the value of 0.96 N obtained in the absence of the magnetic field. Adhesion enhancement is also applicable to lens arrays, as sketched in Figure S12e (Supporting Information) (the enhancement factor in this case is 3.28). The difference in adhesive force and the low adhesion in the absence of the magnetic field make it feasible to grip and release optical elements of various shapes reliably and quickly. Specifically, switching the stiffness of the smart adhesive from low to high facilitates gripping, whereas switching the stiffness from high to low promotes releasing. In addition to being able to adapt to different nonplanar geometries, the smart adhesive is also applicable to misaligned flat glass, i.e., there is an angle *θ* between the smart adhesive and flat glass (Figure S5d, Supporting Information). To demonstrate this point, we compared the adhesive force of the smart adhesive and the bio‐inspired adhesives (with a high‐stiffness backing) on flat glass with various misaligned angles (see Section S8 and Figure S13 and S14, Supporting Information). At *θ* = 2°, the adhesive force of the smart adhesive is reduced by 53% under a magnetic field of 73 mT, while it is reduced by 91.3% for the bio‐inspired adhesives. The results demonstrate that, because of the stiffness modulation, the reduction in adhesion due to misaligned angles is considerably less pronounced for the smart adhesive than for the bio‐inspired adhesives.

Figure 4f demonstrates the extraordinary capabilities of the smart adhesive in manipulating optical elements with diverse shapes, including a 42.7‐g and 10‐cm‐diameter optical dome (Movie S5, Supporting Information), a ball lens with a weight of 670 g and a diameter of 8 cm (Movie S6, Supporting Information), a 7.6‐g concave lens with a radius of curvature of −31.01 mm (Movie S7, Supporting Information), a 12.5‐g convex lens with a radius of curvature of 25.84 mm (Movie S8, Supporting Information), a 3.64‐g and 25.4‐mm diameter meniscus lens with radii of curvature of 32.14 mm (convex side) and −82.2 mm (concave side) (Movie S9 and S10, Supporting Information). More instances of manipulating optical elements with various shapes are illustrated in Figure S15 and Movie S11–S15 (Supporting Information). These examples demonstrate the remarkable capabilities of the smart adhesive in manipulating multiscale (from a few to hundreds of millimeters) optical elements with arbitrary shapes simply by switching on a magnetic field, without the need for complicated visual or tactile feedback.

### 2.5 Application of the Smart Adhesive to the Manipulation and Assembly of Optical Elements

**Figure 5:**
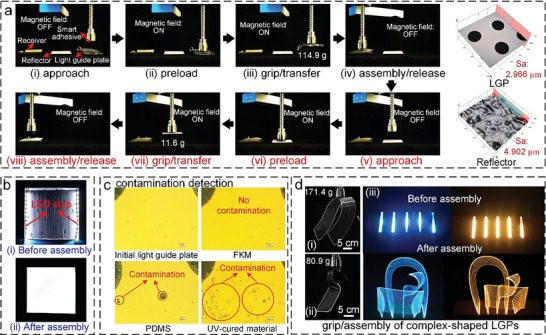
Application of the smart adhesive to the manipulation and assembling of optical elements. a) Snapshots of the assembly process of an ultrathin light box using the smart adhesive, consisting of the gripping, transferring, and releasing of a 114.9‐g LGP and a 11.6‐g reflector sequentially. The surface morphology of the LGP and the reflector is shown on the right; the surface roughness (*Sa*) is 2.966 and 4.902 µm, respectively. b) Comparison of the assembly effect and light diagrams of the ultrathin light box (i) before and (ii) after assembly. c) Detection of the contamination on the LGP surface after assembly: There is no contamination when using the smart adhesive with bio‐inspired FKM adhesives, but considerable contamination (red circles in the images) is left when using the smart adhesive with bio‐inspired adhesives made of PDMS or the UV‐cured material. d) (i) and (ii) Demonstration of the reliable gripping of two extremely complex‐shaped LGPs (171.4 and 80.9 g, respectively) using the smart adhesive and (iii) comparison of the light diagrams of the LGP system before and after assembly.

To explore the potential applications of the smart adhesive to the manipulation and assembly of optical elements, we combined the smart adhesive samples with a transfer robot for the grip/transfer/assembly/release experiments of an ultrathin light box. **Figure** **5a**; Movie S16 (Supporting Information) show the actual assembly process of the ultrathin light box, which includes gripping, transferring, and releasing a 114.9‐g LGP and a 11.6‐g reflector sequentially (the images on the right show the surface morphology; the surface roughness *Sa* is 2.966 and 4.902 µm, respectively). First, with the aid of the preload, the initially soft smart adhesive approaches the LGP until it conforms to it. After applying a magnetic field, the smart adhesive grips the LGP and lifts it up. Then, the smart adhesive transports the LGP to the target position. After alignment and approach, the smart adhesive recovers its initial soft state once the magnetic field is switched off, which allows assembly with the receiver and the release of the LGP. Following the same procedure, the reflector is subsequently transported and assembled with the receiver. The corresponding assembly effect and contamination detection results are illustrated in Figure 5b,c, respectively. Before the assembly, the two light‐emitting diode (LED) strips of the ultrathin light box are point light sources emitting an uneven light (Figure 5b(i)). However, following the assembly, the ultrathin light box emits a uniform light, as illustrated in Figure 5b(ii), due to the combined effects of the LGP and the reflector. In addition, as shown in Figure 5c, the FKM‐based smart adhesive leaves no contamination on the LGP surface. However, the smart adhesives based on PDMS or the UV‐cured material severely contaminate the surface of the LGP (leaving fractured microstructures or residual material) after multiple testing.

In addition to flat LGPs, the unique characteristics of the prepared smart adhesive make it applicable to extremely complex‐shaped LGPs that are difficult to grip using conventional methods, as demonstrated in Figure 5d(i),(ii) (171.4 and 80.9 g, respectively). Figure 5d(iii) shows the actual glowing effects of the LGP system before and after assembly, with the assembly of the LGPs facilitating the uniform divergence of the point light sources. The examples illustrated in Figure 5 for LGPs together with the examples shown in Figure 4; Figure S15 (Supporting Information) for optical elements with various shapes demonstrate the outstanding adaptability of the smart adhesive in reliably and quickly maneuvering optical elements with diverse shapes and sizes without causing surface contamination or damage. We expect that such stiffness‐modulated smart adhesives will become more widespread in intelligent soft robotic systems, providing new opportunities for conventional optical element manipulation technologies.

## 3 Conclusion

In this work, we propose a universal, smart, contact‐adaptive adhesive strategy based on magnetic actuation, which permits the reliable and quick manipulation and assembly of planar or complex‐shaped optical elements with sizes ranging from a few millimeters to tens of centimeters without causing any surface contamination or damage. The smart adhesive is composed of bio‐inspired adhesives on the surface and packaged smart magnetorheological grease. The bio‐inspired adhesives are made of FKM rather than the conventional multicomponent crosslinked polymers, which enables contamination‐free and damage‐free adhesion. The stiffness modulation of the magnetorheological grease based on the magnetorheological effect endows the smart adhesive with a high conformability to arbitrarily shaped optical elements in the soft state, a high interfacial fracture resistance to maintain a high grip force in the stiff state, and the ability to quickly release the optical elements in the recovered soft state. The extraordinary capabilities of the proposed smart adhesive are demonstrated by the contamination‐free and damage‐free gripping, transporting, and releasing of various optical elements, such as convex lenses, concave lenses, ball lenses, and LGPs. This smart adhesive design provides a novel concept for the versatile gripping of optical elements of various shapes, while simultaneously unloading controlling complexities of conventional grippers, which could represent a new approach for the development of switchable adhesives as soft grippers in high‐precision fields, such as the aerospace, medical, and military fields.

However, several aspects should be addressed in future work. The use of weights to release optical elements dramatically increases the release time (particularly for light optical elements), which should be addressed with the use of other ingenious auxiliary devices. Furthermore, more mechanical analyses are required to quantify the contribution of different effects (i.e., van der Waals forces and friction) to the adhesive force of the smart adhesive on various optical elements. In addition, a smaller smart adhesive should be developed to enable the manipulation and assembly of smaller optical elements (with size less than 1 mm).^[^ ^56^, ^57^ ^]^

## 4 Experimental Section

### Materials

Raw FKM (Fluoroelastomer FPM26) was purchased from the Zhonghao Chenguang Research Institute of Chemical Industry Co., Ltd., Zigong, China. Ethyl acetate was supplied by Energy Chemical, Shanghai, China. PDMS (Sylgard 184), with a 1:10 mass ratio of the curing agent to PDMS and the main component being C_6_H_18_OSi_2_, was purchased from Dow Corning. The UV‐cured material (NOA74), with the main component being C_10_H_20_O_2_S, was supplied by NORLAND, USA. The silicone rubber (#600) with an elastic modulus of 0.5 MPa was purchased from Hongye Technology Co., Ltd., Shenzhen, China. The carbonyl iron powder with a particle size of 3.6 µm was purchased from Zhong Hang Zhong Mai Metal Materials Co., Ltd., Suzhou, China. The heat‐resistant flexible glue (Organic silicone glue, HJ‐T326) was supplied by Fangguan Industry Co. Ltd., Shenzhen, China.

### Fabrication of the Smart Adhesive

The smart adhesive was fabricated using an embedding method, which ensures that the design was sufficiently sealed and prevents the magnetorheological grease from leaking during the manipulation process. More details can be found in Section S1 and Figure S1 (Supporting Information).

### Fabrication of the Magnetorheological Grease

To prepare the magnetorheological grease, PDMS base and the carbonyl iron powder in a 1:5 mass ratio were mixed in a beaker for 1 h using a glass rod. The mixture was then degassed for 10 min in a vacuum chamber to obtain the magnetorheological grease. More details can be found in Section S2 and Figure S2 (Supporting Information).

### Surface Analysis via EDS

The EDS point analysis model was employed to detect changes in the element content in areas of possible contamination on the tested glass surfaces. To simplify the analysis, some irrelevant elements, such as Au, H, and S, were not considered. The SEM images and EDS data were obtained using a Hitachi SU8010 scanning electron microscope.

### Measurement of the Adhesive Force

For the adhesion tests of both the bio‐inspired adhesives and the smart adhesive, preload–pause–pulling‐up tests were conducted. Specifically, the test objects (flat glass or optical elements) connected to a load cell by a steel rod were pressed against the bio‐inspired adhesives or the smart adhesive until the preload was reached; they were kept in this state for a while before being pulled up at a fixed speed. The speed for pressing and pulling up was 1 mm·min^−1^, and the pause time was 5 s. It should be emphasized that the adhesion tests for the smart adhesive were conducted under different magnetic fields, whereas no magnetic field was applied in the case of the bio‐inspired adhesives. The magnetic field was applied by an electromagnet. The details of the adhesion test apparatus and the test procedures can be found in Section S4 and Figure S5a,c,d (Supporting Information).

### FEA Simulation

A simplified FEA was conducted using the finite‐element software ABAQUS to simulate the effect of the backing stiffness on the contact and separation processes. The optical element was modelled as a rigid body, while the smart adhesive was modelled as a hyper‐elastic material with a manually adjustable elastic modulus based on the Neo Hooke model. For the simulation of the low modulus, the material constants of the smart adhesive were C_10_ = 0.1, D_1_ = 0.00036, which was approximately equal to 0.6 MPa elastic modulus according to the formula *E* = 6 × C_10_.^[^ ^58^, ^59^ ^]^ For the simulation of the high modulus, the material constants of the smart adhesive were C_10_ = 1, D_1_ = 0.00036, which was approximately equal to 6 MPa elastic modulus. In the simulation, the elastic modulus of the high‐stiffness smart adhesive was set to be 10 times greater than that of the low‐stiffness smart adhesive. The bottom surface of the smart adhesive was fixed, and a contact pair was defined between the upper surface of the smart adhesive and the surface of the optical element. During the contact and separation processes, the optical element was assumed to be pushed and pulled by a preset 0.01‐mm displacement. Then, the smart adhesive interacted with the optical element. Subsequently, the interfacial von Mises stresses of the high‐ and low‐stiffness smart adhesives were analyzed.

## Conflict of Interest

The authors declare no conflict of interest.

## Supporting information

Supporting Information

Supplemental Movie 1

Supplemental Movie 2

Supplemental Movie 3

Supplemental Movie 4

Supplemental Movie 5

Supplemental Movie 6

Supplemental Movie 7

Supplemental Movie 8

Supplemental Movie 9

Supplemental Movie 10

Supplemental Movie 11

Supplemental Movie 12

Supplemental Movie 13

Supplemental Movie 14

Supplemental Movie 15

Supplemental Movie 16

## Data Availability

The data that support the findings of this study are available from the corresponding author upon reasonable request.
