# Supplementary material for: Smart Manipulation of Complex Optical Elements via Contact‐adaptive Dry Adhesives
Source: Adv Sci (Weinh). 2023 Sep 8;10(30):2303874. doi: 10.1002/advs.202303874 (PMC10602548; doi:10.1002/advs.202303874)
Supplement: Supplementary file 1 — Supporting Information [file ADVS-10-2303874-s016.pdf]

ADVANCED SCIENCE
Open Access

Supporting Information

for *Adv. Sci.*, DOI 10.1002/advs.202303874

Smart Manipulation of Complex Optical Elements via Contact-adaptive Dry Adhesives

Shuai Li, Hongmiao Tian, Chunhui Wang, Xiangming Li, Xiaoliang Chen, Xiaoming Chen and Jinyou Shao**

Supporting Information

Smart Manipulation of Complex Optical Elements via Contact-adaptive Dry Adhesives

Shuai Li, Hongmiao Tian, Chunhui Wang, Xiangming Li, Xiaoliang Chen, Xiaoming Chen, Jinyou Shao**

S. Li, H. Tian, C. Wang, X. Li, X. L. Chen, X. M. Chen, J. Shao
Micro- and Nano-technology Research Center, State Key Laboratory for Manufacturing Systems Engineering, Xi'an Jiaotong University, Xi'an, Shaanxi 710049, China
E-mail: hmtian@xjtu.edu.cn

X. Li, X. L. Chen, J. Shao
Frontier Institute of Science and Technology (FIST), Xi'an Jiaotong University, Xi'an, Shaanxi 710049, China
E-mail: jyshao@xjtu.edu.cn

Section S1. Fabrication details of the smart adhesive.

Section S2. Fabrication details of the magnetorheological grease.

Section S3. Fabrication details of the PP mold.

Section S4. Adhesion and contact stiffness characterization details.

Section S5. Comparison of bond energies between carbon-fluorine bonds and other carbon bonds.

Section S6. Influence of the magnetic induction on the shear yield stress of magnetorheological grease.

Section S7. Adhesion characterization of the smart adhesive for optical elements with various shapes.

Section S8. Adaption of the smart adhesive to flat glass with misaligned angles.

Section S9. Gripping demonstrations of optical elements with various shapes.

Figure S1. Fabrication process for the smart adhesive.

Figure S2. Fabrication process of the magnetorheological grease.

Figure S3. Fabrication process of PP mold.

Figure S4. SEM pictures of bio-inspired FKM adhesive microstructures with three radii.

Figure S5. Details of the adhesion and contact stiffness characterization.

Figure S6. Adhesion force test apparatus for the reusability test of the bio-inspired adhesives.

Figure S7. Comparative SEM images before and after 2000 repeated grip and release tests.

Figure S8. Hysteresis loop of the magnetorheological grease.

Figure S9. Viscoelastic response of the magnetorheological grease.

Figure S10. Effect of the dispersed state of the magnetic particles in the magnetorheological grease on its response frequency.

Figure S11. Effect of the dispersed state of the magnetic particles in the magnetorheological grease on its cycle stability.

Figure S12. Adhesion characterization of the smart adhesive for optical elements with various shapes.

Figure S13. Effect of misaligned angles on the adhesive force between bio-inspired adhesives and flat glass.

Figure S14. Effect of misaligned angles on the adhesive force between smart adhesive and flat glass.

Figure S15. Illustration of the proposed smart adhesive reliably holding planar or complex-shaped optical elements ranging from a few millimeters to tens of centimeters.

Table S1. Comparison of bond energies between carbon-fluorine bonds and other carbon bonds.

Movie S1. Magnetorheological effect of the magnetorheological grease with stirring for 1 h.

Movie S2. Magnetorheological effect of the magnetorheological grease with stirring for 1 min.

Movie S3. Cycling test for magnetorheological effect of the magnetorheological grease with stirring for 1 h.

Movie S4. Cycling test for magnetorheological effect of the magnetorheological grease with stirring for 1 min.

Movie S5. Demonstration of the capability of the smart adhesive in manipulating optical dome.

Movie S6. Demonstration of the capability of the smart adhesive in manipulating ball lens.

Movie S7. Demonstration of the capability of the smart adhesive in manipulating concave lens.

Movie S8. Demonstration of the capability of the smart adhesive in manipulating convex lens.

Movie S9. Demonstration of the capability of the smart adhesive in manipulating meniscus lens (convex side).

Movie S10. Demonstration of the capability of the smart adhesive in manipulating meniscus lens (concave side).

Movie S11. Demonstration of the capability of the smart adhesive in manipulating the light guide plate with uniform light guide points on the surface.

Movie S12. Demonstration of the capability of the smart adhesive in manipulating thin glass plate.

Movie S13. Demonstration of the capability of the smart adhesive in manipulating bi-convex lens.

Movie S14. Demonstration of the capability of the smart adhesive in manipulating plano-concave lens.

Movie S15. Demonstration of the capability of the smart adhesive in manipulating plano-convex lens.

Movie S16. Application of the smart adhesive in manipulating and assembling an ultra-thin light box.

Section S1. Fabrication details of the smart adhesive

The fabrication process of the smart adhesive is shown in **Figure S1**. First, a layer of bio-inspired adhesives film made of FKM was prepared. Then, a silicone rubber box with an inner and outer frame was fabricated by cutting the silicone rubber bulk with a thickness of 4 mm, where the silicone rubber bulk was prepared by casting silicone rubber into a mold composed of glass and PET (Polyethylene terephthalate) substrates and subsequent curing. It should be noted that the use of silicone rubber with a 0.5 MPa elastic modulus rather than the more commonly used PDMS with a 2 MPa elastic modulus is to make the packaged smart adhesive's side walls more prone to deformation under the preload, resulting in better conformality with the optical elements. Subsequently, the bio-inspired adhesives film was pasted on the glass and PET substrates, followed by gluing the silicone rubber box onto the bio-inspired adhesives film with a thin spin-coated heat-resistant flexible glue. Then, the silicone rubber box with the bio-inspired adhesives film was placed in a 120□ oven for 10 min to cure the flexible glue to enhance the bonding of the two (>0.74 MPa, much higher than the adhesion strength (~200 kPa) of bio-inspired adhesives film), which ensures the sufficient sealing of the design and prevents the leaking of the magnetorheological grease during the manipulation process. Finally, the magnetorheological grease was poured into the open box, and the partially finished sample was encapsulated with more silicone rubber. After curing and cutting, a finished smart adhesive with a thickness of 4.2 mm was obtained.

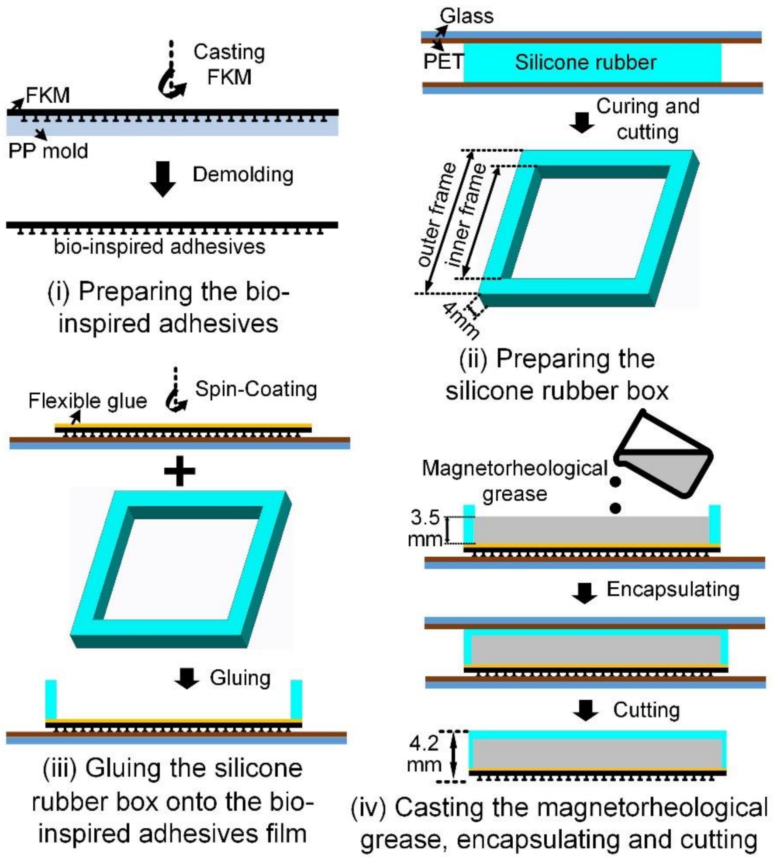

Figure S1. Fabrication process for the smart adhesive. (i) Casting a layer of dissolved FKM on the prepared PP mold, demolding after cross-linking of FKM and evaporation of solvent. (ii) Preparing a 4 mm thick silicone rubber bulk, curing, and cutting into a box with an inner and outer frame. (iii) Gluing the silicone rubber box onto the bio-inspired adhesives film using a thin spin-coated flexible glue. (iv) Casting the magnetorheological grease into the open box, and encapsulating the semi-finished sample with more silicone rubber, followed by cutting after curing to obtain a finished smart adhesive.

Section S2. Fabrication details of the magnetorheological grease

The fabrication process of the magnetorheological grease is shown in **Figure S2**. First, PDMS base and the 3.6 μm carbonyl iron powder with a mass ratio of 1:5 were mixed in a beaker using a glass rod for an hour. Then, the mixture was degassed in a vacuum chamber for 10 min to obtain finished magnetorheological grease. One thing that should be mentioned is that the mass ratio plays a very important role in stiffness modulation effect of the magnetorheological grease. When the mass ratio is less than 1:3, the magnetic responsiveness of the magnetorheological grease is low, resulting in poor stiffness change. If the mass ratio is more than 1:7, the magnetorheological grease will have excessive viscosity and may even become semi-solid, limiting the stiffness modulation effect significantly. We chose the mass ratio of 1:5 to compromise between the magnetic responsiveness and viscosity to achieve a low stiffness at the soft state and a high stiffness at the stiff state, as far as possible.

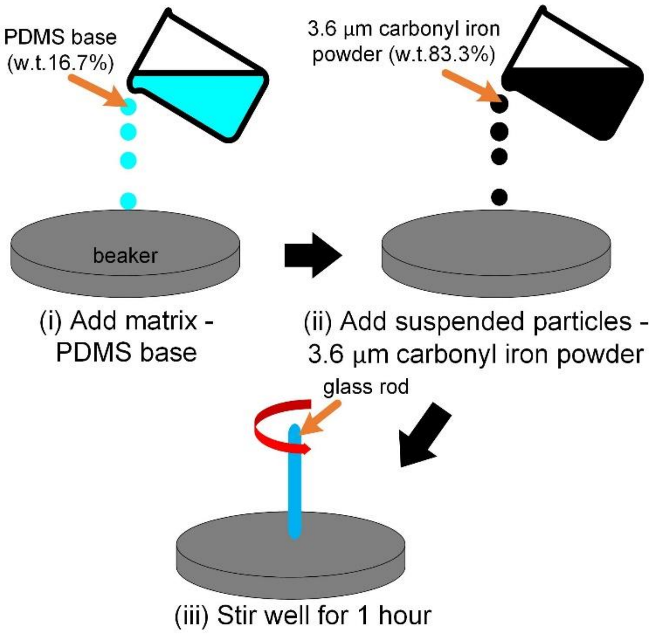

Figure S2. Fabrication process of the magnetorheological grease. (i) Adding 16.7wt% PDMS base into a beaker. (ii) Adding another 83.3wt% 3.6 μm carbonyl iron powder into the beaker. (iii) Stirring well for an hour to obtain finished magnetorheological grease.

Section S3. Fabrication details of the PP mold

The fabrication process of the PP mold is shown in **Figure S3**. First, a block of 5 mm thick bio-inspired adhesives made of PDMS based on our previous research was prepared. Then, a 4 mm thick PP board was placed on a hot plate (230°C). After the PP board was completely melted, the bio-inspired adhesives made of PDMS were pressed on it for 5 min. Finally, after cooling, the PP mold with inverted bio-inspired adhesive structures was obtained by slightly peeling off the bio-inspired adhesives made of PDMS. One thing to note is that the PP material was chosen as a mold because this crystalline plastic is difficult to dissolve in ethyl acetate.

The SEM pictures of bio-inspired FKM adhesive microstructures with three radii (16, 32.5, 40 μm) fabricated using PP molds are shown in Figure S4, exhibiting good uniformity and consistency.

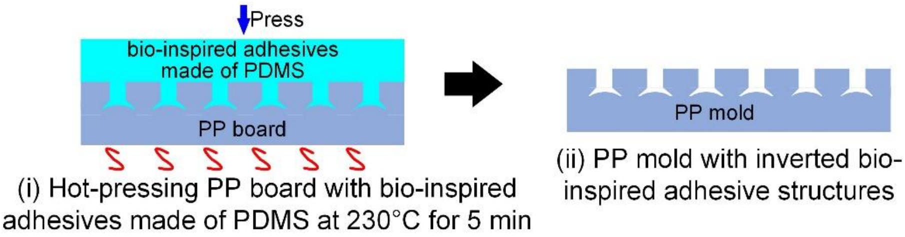

Figure S3. Fabrication process of PP mold. (i) After pressing the bio-inspired adhesives made of PDMS onto the PP board at 230°C for 5 min, (ii) a PP mold is obtained by slightly peeling off the bio-inspired adhesives made of PDMS.

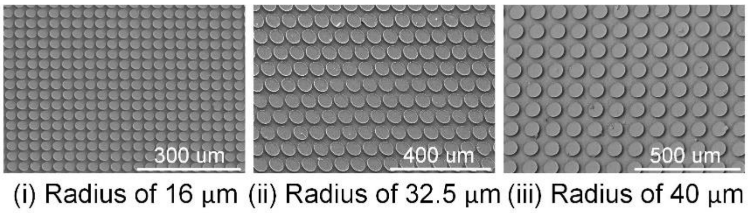

Figure S4. SEM pictures of the bio-inspired FKM adhesive microstructures with three radii. (i) Microstructures with radius of 16 μm. (ii) Microstructures with radius of 32.5 μm. (iii) Microstructures with radius of 40 μm.

Section S4. Adhesion and contact stiffness characterization details

The adhesion force test apparatus and test process for the bio-inspired adhesives are shown in **Figure S5a**. The force test equipment (PT-1198GDP) is supplied by PERFECT Instrument Company, Dongguan, China. A flat glass (5 mm×5 mm) is connected to a load cell with a minimum accuracy of 2 mN through a steel rod. The linear stage of the tester lowers and raises the flat glass in displacement control, and the in-line load cell monitors force throughout the process. A tip/tilt stage is used to facilitate the alignment between the flat glass and the bio-inspired adhesives sample. During the adhesion force test process, the flat glass is pressed against the bio-inspired adhesives sample at a speed of 1 mm/min until the preload is attained, then held for 5 seconds before being pulled up at a speed of 1 mm/min.

The contact stiffness test apparatus and test process for the smart adhesive are shown in Figure S5b. The force test equipment (PT-1198GDP) is supplied by PERFECT Instrument Company, Dongguan, China. A spherical probe (10 mm diameter) is connected to a load cell with a minimum accuracy of 2 mN through a steel rod. The linear stage of the tester lowers and raises the spherical probe in displacement control, and the in-line load cell monitors force throughout the process. A tip/tilt stage is used to facilitate the alignment between the spherical probe and the smart adhesive sample. An electromagnet is used to apply different magnetic induction for the smart adhesive sample (20 mm by 20 mm by 4.2 mm). During the contact stiffness test process, after applying different magnetic induction, the spherical probe is pressed against the smart adhesive sample at a speed of 1 mm/min until the preload is attained, then the contact stiffness is measured using $K=F/\Delta D$ (the preload force F divided by the pressing depth of spherical probe ΔD).

The adhesion force test apparatus and test process for the smart adhesive are shown in Figure S5c. The force test equipment (PT-1198GDP) is supplied by PERFECT Instrument Company, Dongguan, China. The optical elements (convex lenses, concave lenses, ball lens and lens arrays) are connected to a load cell with a minimum accuracy of 2 mN through a steel rod. The linear stage of the tester lowers and raises the optical elements in displacement control, and the in-line load cell monitors force throughout the process. A tip/tilt stage is used to facilitate the alignment between the optical elements and the smart adhesive sample. During the adhesion force test process, the optical elements are pressed against the smart adhesive sample at a speed of 1 mm/min until the preload is attained, after which the magnetic field is triggered and held for 5 seconds before being pulled up at a speed of 1 mm/min.

The test apparatus and test process for the adhesive force of the smart adhesive on flat glass with misaligned angles are shown in Figure S5d. The force test equipment (PT-1198GDP) is

supplied by PERFECT Instrument Company, Dongguan, China. The flat glass (23 mm×23 mm) is connected to a load cell with a minimum accuracy of 2 mN through a steel rod. The linear stage of the tester lowers and raises the flat glass in displacement control, and the in-line load cell monitors force throughout the process. A tip/tilt stage is used to set the misaligned angles between the flat glass and the smart adhesive sample. During the adhesion force test process, the flat glass with misaligned angles is pressed against the smart adhesive at a speed of 1 mm/min until the preload is attained, after which the magnetic field is triggered and held for 5 seconds before being pulled up at a speed of 1 mm/min.

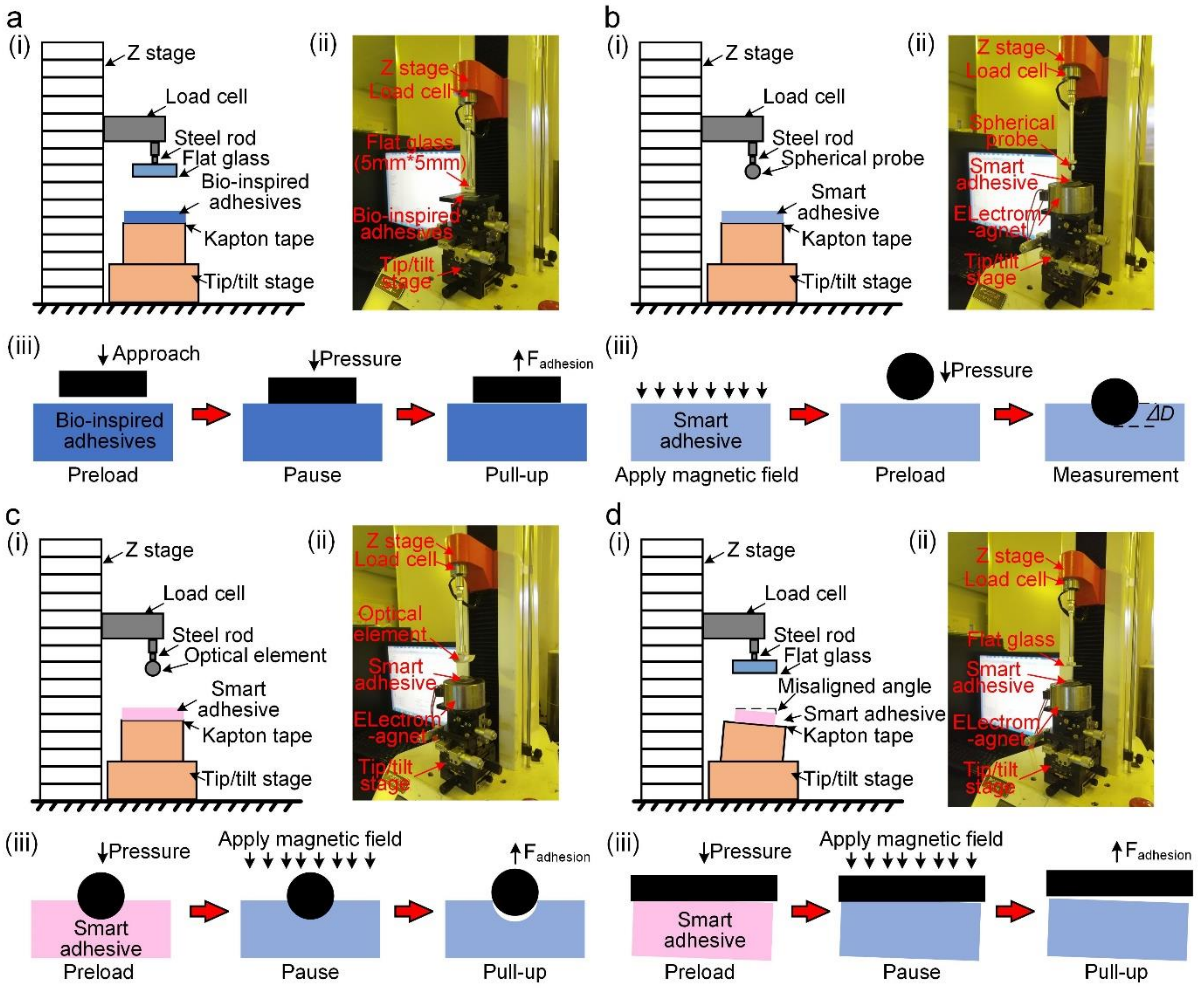

Figure S5. Details of the adhesion and contact stiffness characterization. (a) Adhesion force test apparatus and test process for the bio-inspired adhesives. (i) Schematic and (ii) photography of the adhesion force test apparatus. (iii) Schematic illustration of the preload-pause-pulling up test process for the adhesion force of the bio-inspired adhesives. (b) The contact stiffness test apparatus and test process for the smart adhesive. (i) Schematic and (ii) photography of the contact stiffness test apparatus. (iii) Schematic illustration of the contact stiffness test process for the smart adhesive. (c) Adhesion force test apparatus and test process for the smart adhesive. (i) Schematic and (ii) photography of the adhesion force test apparatus. (iii) Schematic illustration of the preload-pause-pulling up test process for the adhesion force of the smart

adhesive. (d) Test apparatus and test process for the adhesive force of the smart adhesive on flat glass with misaligned angles. (i) Schematic and (ii) photography of the adhesion force test apparatus. (iii) Schematic illustration of the preload-pause-pulling up test process for the adhesion force of the smart adhesive on flat glass with misaligned angles.

The adhesion force test apparatus for the reusability test of the bio-inspired adhesives is shown in Figure S6. The force test equipment (ESM303) with a 50 Hz sampling frequency is supplied by Mark-10, USA. A flat glass (5 mm×5 mm) is connected to a load cell with a minimum accuracy of 5 mN through a steel rod. The linear stage of the tester lowers and raises the flat glass in a displacement-controlled manner for 2000 repeating cycles, and the in-line load cell monitors force throughout the process. A tip/tilt stage is used to facilitate the alignment between the flat glass and the bio-inspired adhesives sample. During the adhesion force test process, the flat glass is pressed against the bio-inspired adhesives sample at a speed of 10 mm/min until the preload is attained, and then pulled up at a speed of 10 mm/min.

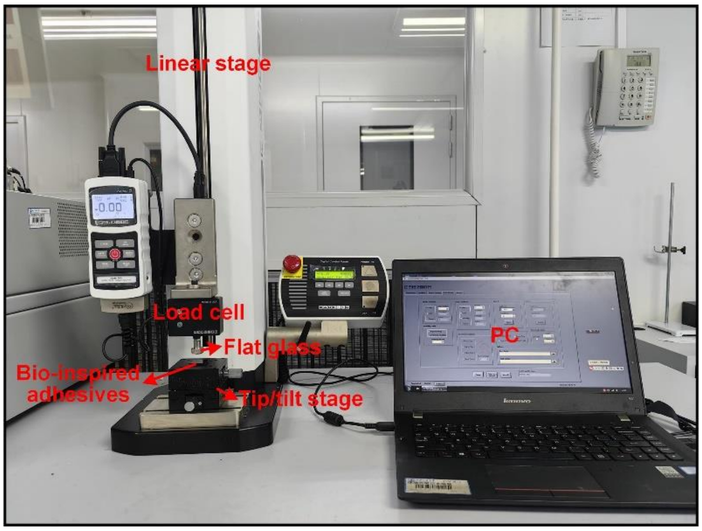

Figure S6. Adhesion force test apparatus for the reusability test of the bio-inspired adhesives. Photography of the adhesion force test apparatus (ESM303, Mark-10, USA).

The scratch resistance of the prepared biomimetic FKM layer is also outstanding, and no damage, scratch or fracture is observed on the surface of the structure after 2000 repeated grip and release tests using the apparatus in Figure S6, as shown in the comparative SEM images before and after the tests in Figure S7.

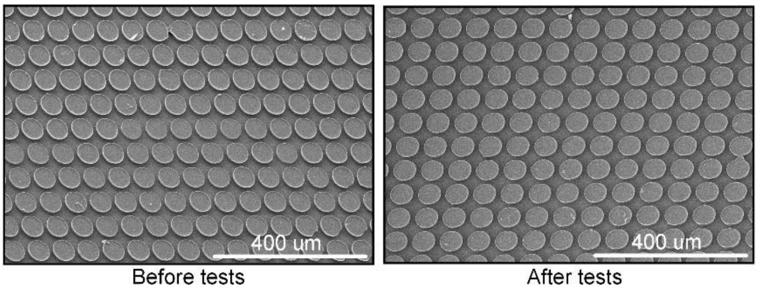

Figure S7. Comparative SEM images before and after 2000 repeated grip and release tests.

Section S5. Comparison of bond energies between carbon-fluorine bonds and other carbon bonds

One reason for the extraordinary fatigue durability and contamination-free adhesion of bio-inspired adhesives made of FKM is that the C–F bond energy of FKM is higher (485 KJ/mol) than other carbon bond energies (table S1).

Table S1. Comparison of bond energies between carbon-fluorine bonds and other carbon bonds.

Compound	Chemical bond	Bond energy/(KJ/mol)
**F_3CF**	C–F	485
**$-F_2C-H$**	C–H	431
**Hydrocarbon**	C–H	413
**Hydrocarbon**	C–O	351
**Hydrocarbon**	C–C	348

Section S6. Influence of the magnetic induction on the shear yield stress of magnetorheological grease

According to Bingham fluid model, to enable magnetic chains to slip or bend, the applied shear stress τ must be higher than the shear yield stress τ_y of magnetorheological grease as per the Eq. (1).[53]

$$\tau=\tau_y \cdot \mathrm{sgn}\left(\dot{\gamma}\right)+\eta \cdot \dot{\gamma} \qquad (1)$$

where, τ is the applied shear stress, τ_y is the shear yield stress of magnetorheological grease, η is the plastic viscosity of magnetorheological grease, $\dot{\gamma}$ is the shear rates. The shear yield stress τ_y reflects the shearing stiffness of magnetorheological grease, with the following dependence on magnetic induction according to Fang's research: [52]

$$\tau_y = \alpha H^2 \left(\frac{\tanh \sqrt{H/H_c}}{\sqrt{H/H_c}} \right) \qquad (2)$$

where, α is related to the susceptibility of the magnetorheological grease and volume fraction or other analogous physical parameters, H represents the magnetic field, H_c represents the critical magnetic field for magnetorheological grease.

τ_y possesses two limiting behaviors with respect to H:

$$\tau_y = \alpha H^2 \text{ for } H \ll H_c \qquad (3)$$

$$\tau_y = \alpha H^{3/2} \sqrt{H_c} \text{ for } H \gg H_c \qquad (4)$$

At low H, τ_y is proportional to H^2, due to the local saturation of the magnetized particles, while $\tau_y \propto H^{3/2}$ at intermediate range of H. For this work, applied magnetic field H ($\leq$ 73 mT) is far below H_c, thus τ_y is proportional to H^2.

The hysteresis loop of the magnetorheological grease tested by MPMS is shown in Figure S8. The sample weight is 55.28 mg. The magnetorheological grease can exhibit excellent magnetization properties with 123.46 A·m^2/kg magnetization at 800.83 kA/m magnetic field strength, which is capable of inducing high shear yield stress. The coercive force and remanence of the magnetorheological grease are 0.8528 kA/m and -0.66269 A·m^2/kg, respectively, demonstraing that the hysteresis of the magnetorheological grease is very weak.

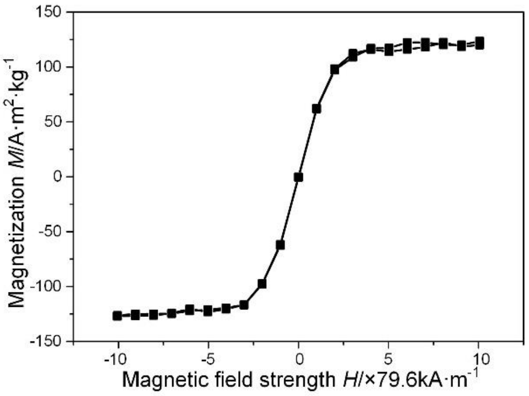

Figure S8. Hysteresis loop of the magnetorheological grease.

Figure S9 shows the viscoelastic response of the magnetorheological grease tested by MCR302. It can be seen that the viscosity of the magnetorheological grease exhibits an obvious shear thinning from 14200 Pa·s at 0.1 s^{-1} shear rate to 0.0106 Pa·s at 1000 s^{-1} shear rate (Figure S9a) (Inset: Magnified image of the viscosity response over the shear rate range of 0.1 s^{-1} to 104 s^{-1}). In addition, at a constant frequency of 5 Hz, the magnetorheological grease behaves similarly to a viscous liquid because its loss modulus is much higher than its storage modulus at various strains.

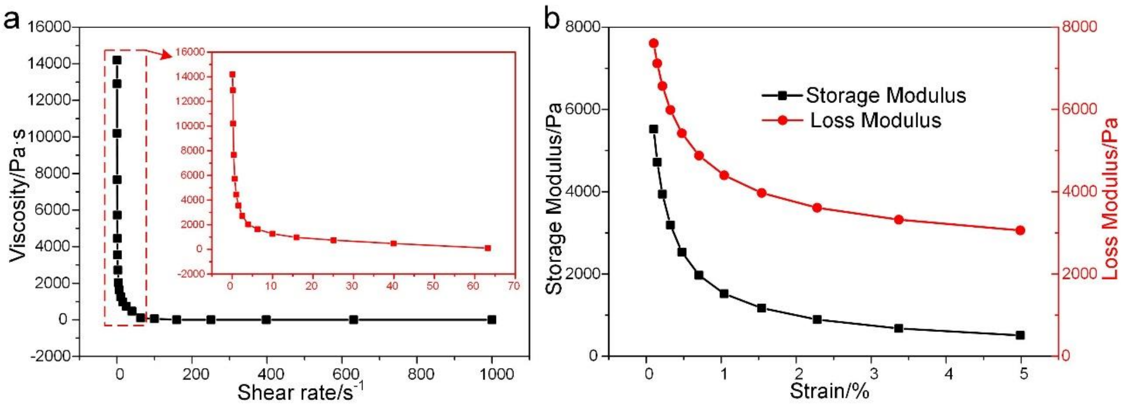

Figure S9. Viscoelastic response of the magnetorheological grease.

The effect of the dispersed state of the magnetic particles in the magnetorheological grease on its response frequency and cycle stability is also discussed, as shown in Figures S10 and S11. After applying a magnetic field of 200 mT by a permanent magnet, it is obvious that the magnetorheological effect of the magnetorheological grease with stirring for 1 h is slightly better than that with stirring for 1 min at the same response time from Figure S10 due to the better dispersed state of the magnetorheological grease with stirring for 1 h (Movies S1 to S2). Using the apparatus in Figure S6, 10 cycling tests of the magnetorheological effect of the magnetorheological grease are performed. The speed of the apparatus is set to the maximum, i.e. 1100 mm/min, and one cycle time is about 16 s. The magnetic inductions close to and

away from the permanent magnet are 200 and 4 mT, respectively. As shown in Figure S11, the magnetorheological effect of the magnetorheological grease with stirring for 1 h is always stable (Movie S3). However, the magnetorheological effect of the magnetorheological grease with stirring for 1 min perfroms poorly in the first few cycles and better and better in the last few cycles due to the more uniform dispersed state induced by the magnetorheological effect (Movie S4).

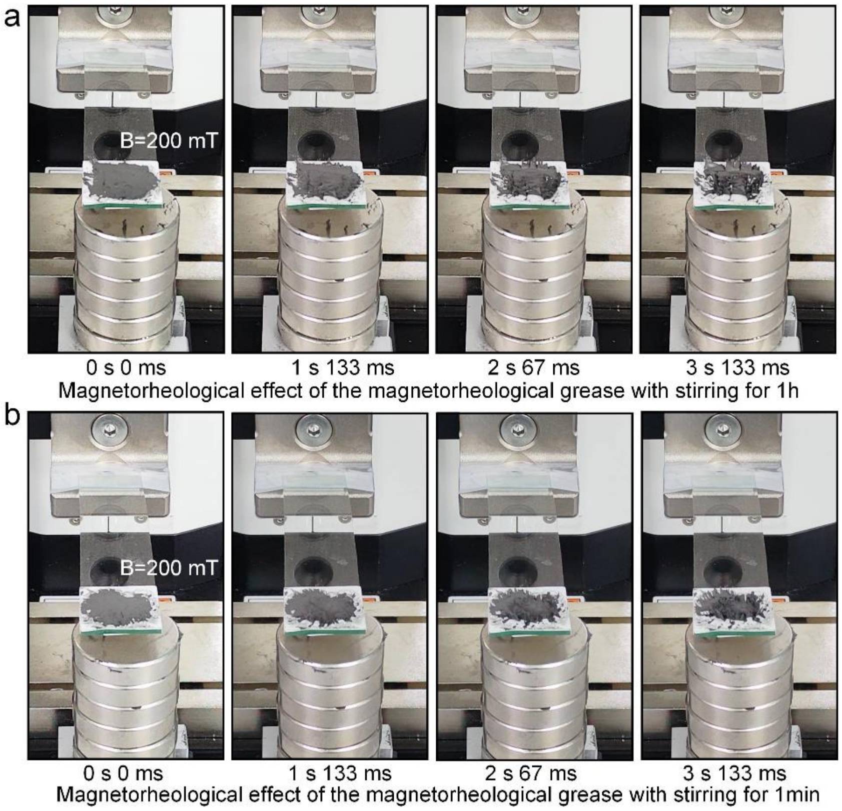

Figure S10. Effect of the dispersed state of the magnetic particles in the magnetorheological grease on its response frequency.

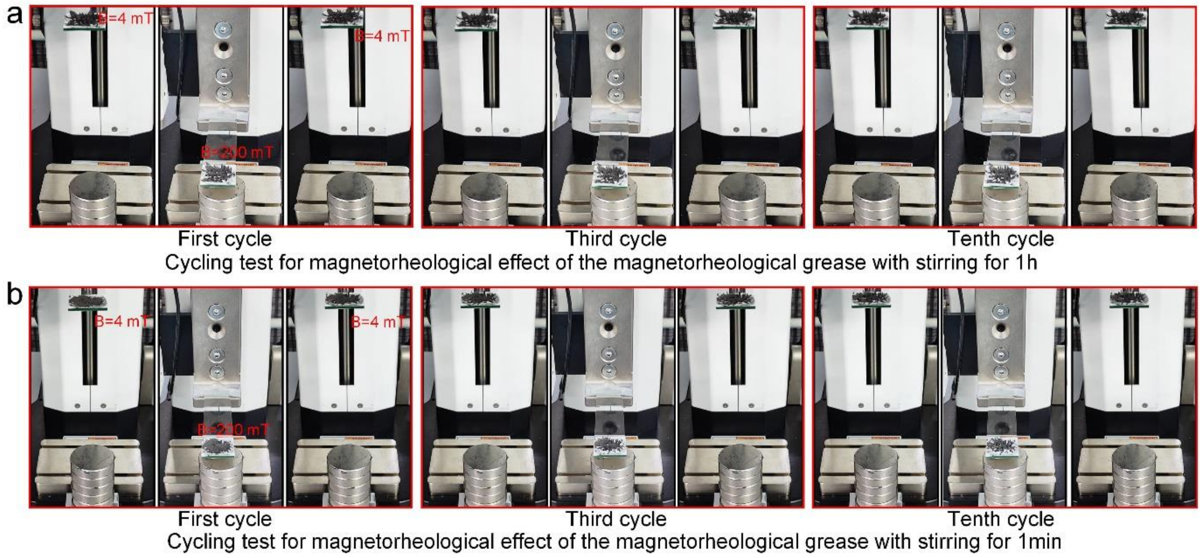

Figure S11. Effect of the dispersed state of the magnetic particles in the magnetorheological grease on its cycle stability.

Section S7. Adhesion characterization of the smart adhesive for optical elements with various shapes

The force-time curves obtained for the smart adhesive (20 mm by 20 mm by 4.2 mm) on No. 1 convex lens (inset, curvature radius of 13.13 mm) under increasing magnetic field and a 2-N preload force are shown in **Figure S12a**, where the magnetic field was only applied during the pause and pull-up stages because the preload stage requires a high conformality of the smart adhesive to the convex lens. Clearly, as the magnetic induction increases, so does the adhesion force.

Influence of the varying magnetic induction on the adhesion force for No.4' concave lens (inset, curvature radius of -77.52 mm) is illustrated in Figure S12b. At a 2-N preload, the adhesion force increases with the increase of the magnetic induction.

The influence of magnetic induction on the adhesive force for an 8-cm spherical lens is also investigated. It is obvious that as the preload increases, the adhesive force increases under the same magnetic induction (0 mT or 73 mT). At a 2.5-N preload, the adhesive force under a 73 mT magnetic induction is as high as 7.25 N, which is more than 6.55 times the 0.96 N obtained in the absence of a magnetic field (Figure S12c). In addition, at a 2-N preload, as the magnetic induction increases, the adhesion force increases as well, as shown in Figure S12d.

The smart adhesive is not only adaptable to individual optical elements, but also to lens arrays, as illustrated in Figure S12e, which shows the effect of magnetic induction on the adhesive force for a lens array (peak-to-valley distance, 1.08 mm; center-to-center distance, 6.08 mm). Under the same magnetic induction, as the preload increases, the adhesive force increases as well. However, since the embedding depth of the smart adhesive into the lens array is limited by the peak-to-valley distance on the lens array, the adhesion force lastly becomes stabilized. At a 6-N preload, the adhesive force under 73 mT magnetic induction can reach 1.07 N, which is up to 4.28 times the 0.25 N in the absence of a magnetic field.

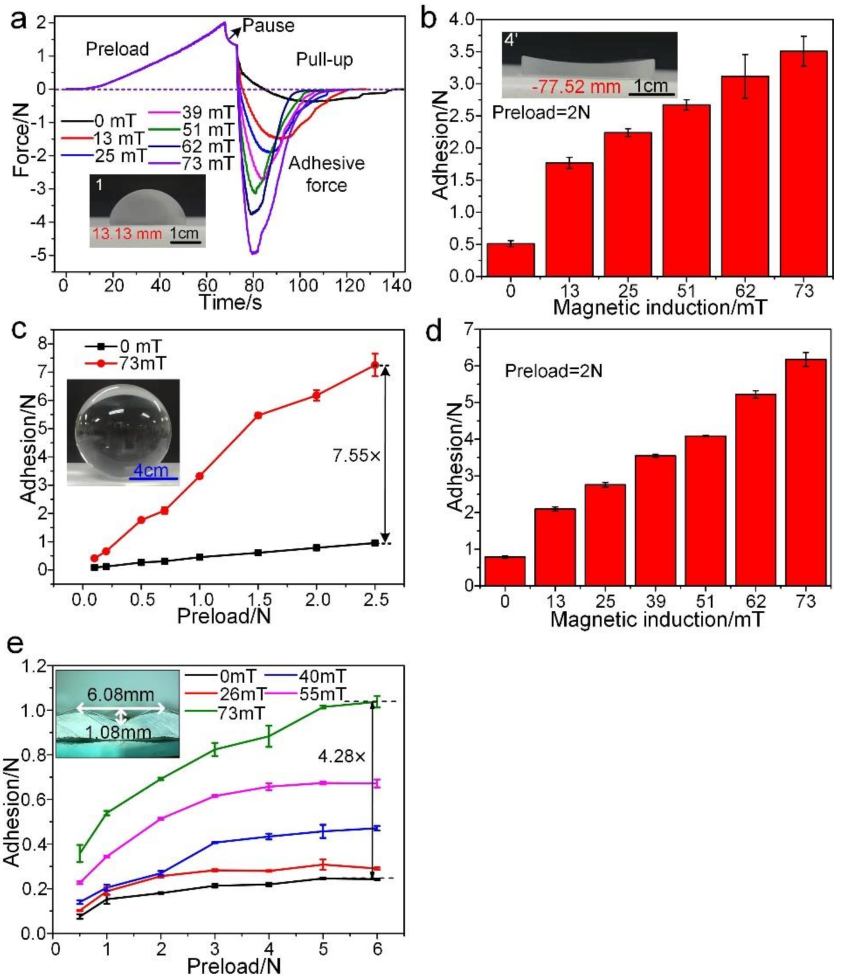

Figure S12. Adhesion characterization of the smart adhesive for optical elements with various shapes. (a) Force-time curves of the smart adhesive (20 mm by 20 mm by 4.2 mm) testing process on No. 1 convex lens (inset, curvature radius of 13.13 mm) under increasing magnetic induction and a 2-N preload force. (b) Influence of the magnetic induction on the adhesive force for No. 4' concave lens (inset, curvature radius of -77.52 mm) at a 2-N preload. (c) Comparison of the adhesive force for a spherical lens with a diameter of 8 cm (inset) under 0 and 73 mT magnetic induction (as functions of the preload). (d) Influence of the magnetic induction on the adhesive force for the spherical lens with a diameter of 8 cm at a 2-N preload. (e) Comparison of the adhesive force for a lens array (inset, peak-to-valley distance, 1.08 mm; center-to-center distance, 6.08 mm) under varying magnetic induction (as functions of the preload). Error bars denote ±SD.

Section S8. Adaption of the smart adhesive to flat glass with misaligned angles

To demonstrate the adaptability of the smart adhesive to flat glass with misaligned angles, we compared the adhesive force of the smart adhesive and bio-inspired adhesives (with a high stiffness backing) on flat glass with misaligned angles.

Figure S13a shows snapshots of misaligned angles (0°, 0.5°, 1°, 1.5°, and 2°) between flat glass and bio-inspired adhesives in the test processes. The force-time curves of bio-inspired adhesives testing processes on flat glass under varying misaligned angles and a 3-N preload force are shown in Figure S13b. As the misaligned angle grows, the contact area between bio-inspired adhesives and flat glass decreases, reducing the adhesion force sharply. The adhesion force at 2° misaligned angle is reduced by 91.3% compared to that of 0° (Figure S13c).

Figure S14a shows snapshots of misaligned angles (0°, 1°, 2°, 3°, and 5°) between flat glass and smart adhesive in the test processes. At 0° misaligned angle, the smart adhesive's adhesive force as a function of the preload under 0 and 73 mT magnetic induction on flat glass is illustrated in Figure S14b, showing that the adhesive force increases with the preload for identical magnetic induction. Figure S14c illustrates the influence of misaligned angles on the adhesive force between smart adhesive and flat glass under 0 and 73 mT magnetic induction. It demonstrates that due to the stiffness modulation of the smart adhesive, the reduction of adhesive force between the smart adhesive and flat glass is less than the reduction of adhesive force between bio-inspired adhesives and flat glass with the increase of misaligned angle. When compared to 0° misaligned angle, the adhesion force under 0 and 73 mT magnetic induction at 2° misaligned angle reduced by 79.7% and 53%, respectively.

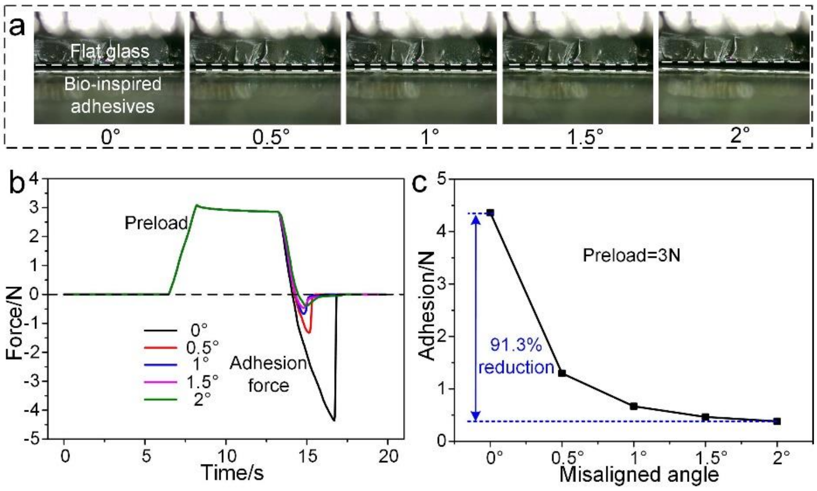

Figure S13. Effect of misaligned angles on the adhesive force between bio-inspired adhesives and flat glass. (a) Snapshots of misaligned angles (0°, 0.5°, 1°, 1.5°, and 2°) between flat glass and bio-inspired adhesives in the test processes. (b) Force-time curves of bio-inspired adhesives testing processes on flat glass under varying misaligned angles and a 3-N preload force. (c) Influence of the misaligned angles on the adhesion force at a 3-N preload.

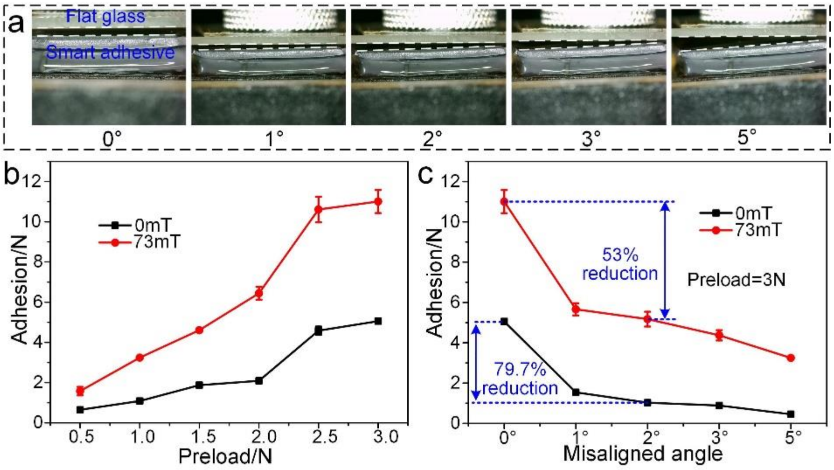

Figure S14. Effect of misaligned angles on the adhesive force between smart adhesive and flat glass. (a) Snapshots of misaligned angles (0°, 1°, 2°, 3°, and 5°) between flat glass and smart adhesive in the test processes. (b) Smart adhesive's adhesive force as a function of the preload for flat glass with 0° misaligned angle under 0, 73 mT magnetic induction. (c) Influence of misaligned angles on the adhesive force between smart adhesive and flat glass under 0 and 73 mT magnetic induction at a 3-N preload.

Section S9. Gripping demonstrations of optical elements with various shapes

We combined the smart adhesive samples with a transfer robot (AH-5020-054S, QKM Technology (Dong Guan) Co., Ltd) to demonstrate the extraordinary capabilities of the smart adhesive in gripping optical elements with diverse shapes and sizes. More examples are illustrated in Figure S15, including a 75.8 g light guide plate with uniform light guide points on the surface (inset); a 50.5 g thin glass plate with a diameter of 25.4 cm and a thickness of 400 μm; a 23.4 g bi-convex lens with a radius of curvature of 52.45 mm; a 1.14 g plano-concave lens with a radius of curvature of -15.45 mm; a 0.71 g plano-convex lens with a radius of curvature of 25.75 mm.

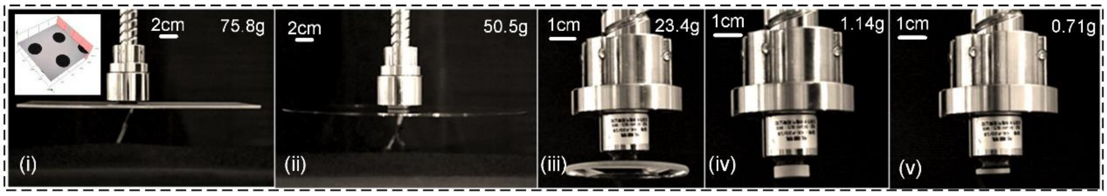

Figure S15. Illustration of the proposed smart adhesive reliably holding planar or complex-shaped optical elements ranging from a few millimeters to tens of centimeters. (i) a light guide plate; (ii) a thin glass plate with a thickness of 400 μm; (iii) a bi-convex lens; (iv) a plano-concave lens; (v) a plano-convex lens.
